# Increase In Il-31 Serum Levels Is Associated With Reduced Structural Damage In Early Axial Spondyloarthritis

**DOI:** 10.1038/s41598-018-25722-z

**Published:** 2018-05-16

**Authors:** Nicolas Rosine, Adrien Etcheto, Houria Hendel-Chavez, Raphaèle Seror, Karine Briot, Anna Molto, Philippe Chanson, Yassine Taoufik, Daniel Wendling, Rik Lories, Francis Berenbaum, Rosaline van den Berg, Pascal Claudepierre, Antoine Feydy, Maxime Dougados, Christian Roux, Corinne Miceli-Richard

**Affiliations:** 1 Paris Descartes University, Department of Rheumatology - Hôpital Cochin. Assistance Publique - Hôpitaux de Paris, EULAR center of excellence, Paris, France; 2 INSERM (U1153) Clinical epidemiology and biostatistics, PRES Sorbonne Paris-Cité, Paris, France; 3 Service d’immunologie, Assistance Publique-Hôpitaux de Paris (AP-HP), Hôpital Bicêtre, Le Kremlin Bicêtre, France; 4 Service de rhumatologie, Université Paris-Sud, Assistance Publique-Hôpitaux de Paris (AP-HP), Hôpitaux universitaires Paris-Sud, Center of Immunology of Viral Infections and Autoimmune Diseases (IMVA), Institut pour la Santé et la Recherche Médicale (INSERM) U1184, Le Kremlin Bicêtre, Paris, France; 5 Service d’endocrinologie, Assistance Publique-Hôpitaux de Paris (AP-HP), Hôpital Bicêtre, Le Kremlin Bicêtre, France; 6 Service de Rhumatologie, CHRU de Besançon et Université de Franche Comté, Besançon, France; 7 Laboratory of Tissue Homeostasis and Disease, Skeletal Biology and Engineering Research Center, Department of Development and Regeneration, KU Leuven, Belgium; 8 University Pierre & Marie Curie and Inserm, DHU i2B, AP-HP, Hospital Saint-Antoine, Paris, France; 9 Department of Rheumatology, Erasmus University Medical Center, Rotterdam, The Netherlands; 10 Service de rhumatologie, Assistance Publique-Hôpitaux de Paris (AP-HP), Hôpital Henri-Mondor, Université Paris Est Créteil, E 7379-EpidermE, Créteil, France; 11 Paris Descartes University, Department of Radiology - Hôpital Cochin. Assistance Publique - Hôpitaux de Paris, Paris, France; 12 Unité Mixte AP-HP/Institut Pasteur, Institut Pasteur, Immunoregulation Unit, Paris, France

## Abstract

In spondyloarthritis, little is known about the relation between circulating cytokines and patient phenotype. We have quantified serum levels of T helper type 1 cell (Th1), Th2 and Th17 cytokines in patients with recent-onset axial spondyloarthritis (AxSpA) from the DESIR cohort, a prospective, multicenter French cohort consisting of 708 patients with recent-onset inflammatory back pain (duration >3 months but <3 years) suggestive of AxSpA. Serum levels of Th1, Th2, and Th17 cytokines were assessed at baseline in patients from the DESIR cohort fulfilling the ASAS criteria (ASAS+) and were compared with age- and sex-matched healthy controls. At baseline, ASAS+ patients (n = 443) and healthy controls (n = 79) did not differ in levels of most of the Th1, Th2 and Th17 cytokines except for IL-31, and sCD40L, which were significantly higher for ASAS+ patients than controls (p < 0.001 and p = 0.012, respectively). On multivariable analysis of ASAS+ patients, IL-31 level was associated with sCD40L level (p < 0.0001), modified Stoke AS Spine Score (mSASSS) < 1 (p = 0.035). The multivariable analyses showed that IL-31 was an independent factor associated with mSASSS < 1 (p = 0.001) and low bone mineral density (p = 0.01). Increased level of IL-31 might protect against structural damage but is also related to low BMD.

## Introduction

Axial spondyloarthritis (AxSpA) is characterized by inflammation, followed by bone erosion and syndesmophyte formation in the more severe forms of the disease. These structural damages in AxSpA remain a major challenge in clinical practice. Indeed, no treatment used in daily practice have been reproductibly shown to stop syndesmophyte formation despite their capacity to significantly reduce inflammation and the painful manifestations of the disease^1–3^. Bone formation involves cellular actors, growth factors (bone morphogenetic protein), and signaling pathways (Wnt and its inhibitors DKK-1 and sclerostin), whose activity is modulated by pro-inflammatory cytokines, in particular tumor necrosis factor (TNF). The involvement of these different actors and activators can vary depending on the evolutionary stages of the disease.

The T helper type 1 (Th1) group of cytokines including interferon γ (IFNγ) cells have been implicated in the pathogenesis of SpA^4^. However, this key role of Th1 cytokines has been challenged by the identification of interleukin-23 (IL-23), a member of the IL-12 family of heterodimeric cytokines, and the IL-17 family cytokines as key players in SpA pathogenesis. The finding is supported by studies showing higher frequency of IL-17–producing cells in peripheral blood from patients than healthy donors^5–10^. However, Th1 and Th17 cytokines may not be the only cytokine subsets to promote inflammation during the course of the disease.

Some data have shown that the Th2 pathway may be involved in the pathophysiology of SpA. To gain further insight into the role of the Th1/Th2 cytokine balance in patients with AxSpA, Rudwaleit *et al*. studied the impact of Th2-driven atopy in AxSpA versus rheumatoid arthritis (RA) patients^11^. Atopy was associated with decreased disease severity in RA but this was not seen in AxSpA.

To gain more insight into the role of several cytokines in the disease phenotype and their potential role in the structural severity of AxSpA, we measured a broad range of Th1, Th2 and Th17 cytokines in patients at an early stage of the disease.

## Patients and Methods

### Study population: The DESIR cohort

DESIR is a longitudinal prospective cohort of patients with inflammatory back pain (IBP) suggestive of axSpA of recent onset recruited from 25 regional centres in France^12^. All participants in the study gave their written informed consent and all methods were carried out in accordance with relevant guidelines and regulation. A detailed description of the centres, organisation of the cohort and full detailed protocol are available at http://www.lacohortedesir.fr (ClinicalTrials.gov: NCT01648907 approved by Comité de Protection des Personnes Île de France III reference CPP file N°Am5975-5-2457). The participants included in the DESIR cohort were >18 and <50 years old. IBP was defined according to the Calin and/or Berlin criteria^13,14^, lasting for >3 months but <3 years, with symptoms suggestive of AxSpA according to the local rheumatologist’s assessment (i.e., score ≥5 on a numerical rating scale of 0–10, with 0, not suggestive, and 10, very suggestive of AxSpA). The exclusion criteria were other clearly defined spinal disease (e.g., discarthrosis), history of any biologic treatment and history or current disorders that might interfere with the validity of the informed consent and/or prevent optimal compliance with the cohort. Corticosteroid intake was permitted only with doses of <10 mg prednisone per day and had to be stable for at least 4 weeks before baseline. A total of 708 patients with IBP were included between October 2007 and April 2010. Patients were evaluated every 6 months during the first 2 years and then on a yearly basis for an expected total follow-up duration of 10 years.

The following data were collected at the baseline visit:

Clinical data were axial involvement, peripheral joint involvement, uveitis, inflammatory bowel disease, psoriasis, enthesitis, dactylitis, synovitis, duration of symptoms (defined as the time between the fist axial symptom and the initial interview), activity and severity features of the disease. Other data collected were Bath Ankylosing Spondylitis Global Assessment (BAS-G) (0–100); Bath Ankylosing Spondylitis Disease Activity Index (BASDAI) (0–100); Bath Ankylosing Spondylitis Functional Index (BASFI) (0–100); spinal mobility as measured by the Bath Ankylosing Spondylitis Metrology Index (BASMI) (0–10); Medical Outcomes Survey short form 36 score (SF-36); Health Assessment Questionnaire (HAQ); and the use of non-steroidal anti-inflammatory drugs (NSAIDs) or disease-modifying antirheumatic drugs (DMARDs) (yes/no).

To have a homogeneous population, patients were classified by the Assessment of Spondyloarthritis International Society (ASAS) criteria^15^, the B. Amor (AMOR) or European Spondylarthropathy Study Group (ESSG) criteria. In our study, only those patients who fulfilled these classification criteria were assessed within the DESIR cohort. Risk factors for osteoporosis collected were age, gender, menopause, tobacco use, excess alcohol consumption, height, weight, body mass index (BMI; kg/m2), and the presence of inflammatory bowel disease.

Biological parameters: erythrocyte sedimentation rate (ESR), levels of C-reactive protein (CRP) and high-sensivity CRP (hsCRP) and presence of human leukocyte antigen B27 (HLA-B27). The ankylosing spondylitis disease activity score (ASDAS)–CRP was calculated by using CRP level^15^.

Imaging modalities and central reading of x-ray and MRI images were described elsewhere^16,17^. In brief, pairs of trained central readers independently evaluated x-ray and MRI images of both the sacroiliac joints and spine, with blinding to clinical data and the other imaging modalities. For dichotomus scores, if the 2 readers disagreed, an adjudicator’s score was used. Scores for 2 of 3 agreeing readers were used. For continuous scores, the mean of the 2 agreeing readers was used.

Based on the modified New York (mNY) criteria, sacroiliitis was defined as at least grade >2 bilaterally or grade 3–4 unilaterally by central reading (pos-X-SI)^18^. Lumbar and cervical spine radiographs were scored by using the modified Stoke AS Spine Score (mSASSS)^19^. T1-weighted fast spin echo and short τ inversion recovery 1–1.5 tesla MRI of the whole spine (MRI-spine) and sacroiliac joints (MRI-SI) was performed to assess inflammatory and structural changes at baseline. MRI-SI images were considered positive according to the ASAS definition (i.e., presence of bone marrow edema [BME] lesions highly suggestive of SpA (with ≥1 BME lesion on ≥2 consecutive slices or several BME lesions visible on a single slice)^20^. The MRI inflammation in the spine was defined according to the ASAS criteria^21^.

Bone mineral density (BMD) measurements: BMD was measured by dual-energy x-ray absorptiometry at baseline for all included patients in 12 centres (i.e., half of the participating centres) with investigators having expertise in BMD measurements. Experienced investigators obtained BMD measurements with Hologic, Inc. or Lunar (GE Healthcare) devices. BMD of the lumbar spine (second to fourth vertebrae) and the upper part of the left femur (total femur and femoral neck) was determined. The results are given as BMD (g/cm2), Z- and T-scores. The definition of low BMD in young adults lacks consensus. The International Society of Clinical Densitometry recommends the threshold of Z-score −2 SD for defining low BMD^22^; the WHO definition, based on T-scores, cannot be used for non-menopausal women and men <50 years old^22^. Therefore, we used Z-score ≤ −2 SD (at least one site) as low BMD. One site was defined by total lumbar spine (L1–L4), or total hip, or femoral neck. Z-scores were determined according to references provided by the manufacturers. Gender-specific Z-scores were based on female and male reference curves. All examinations were performed according to the manufacturer’s recommendations. Devices were controlled by measuring a spine phantom at least 3 times a week throughout the study.

### Healthy controls: Variété cohort

Patients were compared to 79 age - and sex-matched healthy controls from the “Variété cohort”.

Variété is an open, prospective, French national, multicenter, non-randomized study of healthy volunteers^23^ (ClinicalTrials.gov: NCT01831648). Subjects included in this cohort were a large random selection from the general population that included representation from all age groups (about 100 participants for each decade age range). Participants with medical conditions and receiving medications that may affect cytokines measurement were excluded. A total of 974 healthy participants were recruited in 10 centers in France. Each participant underwent clinical examination^23^. Personal medical history was recorded and gonadal status evaluated. Patients underwent biological standard workup, and 80 ml blood was sampled; serum and plasma samples were aliquoted and frozen and stored at −80 °C before hormone measurements. All patients gave their informed consent to participate in the study, which was approved by the local ethics committee.

### Serum assays

The biological resources centre (Paris, Bichat Hospital Claude Bernard–CRB BCB, Certificate no. 34457, S. Tubiana) was in charge of centralising and managing biological data collection.

Cytokines in serum samples were measured by using the Bio-Plex Pro Human Th17 Cytokine Assays 1 (Bio-Rad Laboratories, Inc.). The samples were diluted 4-fold with the diluting solution and centrifuged at 10,000 g for 5 min. An amount of 50 μL of the supernatant was used for the cytokine assay in accordance with the manufacturer’s instructions. The following cytokines were measured: IL-1β, IL-4, IL-6, IL-10, IL-17A, IL-17F, IL-21, IL-22, IL-23, IL-25, IL-31, IL-33, IFNγ, soluble CD40 ligand (sCD40L), and TNFα. The lower limits of detection according to our standard curves were 0.24 pg/ml for IL-1β, 1.33 pg/ml for IL-4, 1.65 pg/ml for IL-6, 1.99 pg/ml for IL-10, 1.20 pg/ml for IL-17A, 3.04 pg/ml for IL-17F, 8.97 pg/ml for IL-21, 3.88 pg/ml for IL-22, 7.35 pg/ml for IL-23, 1.00 pg/ml for IL-25, 3.87 pg/ml for IL-31, 4.18 pg/ml for IL-33, 2.54 pg/ml for IFNγ, 2.41 pg/ml for sCD40L, and 0.57 pg/ml for TNFα.

To define the normal range of cytokine measurements, 50 μL of serum from the 79 healthy controls was provided.

DKK-1 level was quantified by sandwich ELISA (BiomedicaMedizinprodukte, Vienna, Austria). Quality controls for DKK-1 measurements have been provided elsewhere^24^.

### Statistical analysis

Statistical analyses were performed on the database locked on July 2014. Cytokines in serum are presented in percentages of samples with detectable cytokine (percent detectable, %) and as cytokine level (mean ± SD, pg/ml) for each group. Cytokines with values below the limits of detection were graded as negative and the levels were assigned a numerical value of 0 pg/ml for statistical analysis. Spearman correlation was used to examine the relation between IL-31 level and that of other cytokines. To evaluate the characteristics of the axSpA patients associated with IL-31 serum level, we performed a first-step univariate analyses including the variables age, disease duration, sex, axial and peripheral involvement, tobacco use, CRP and hsCRP levels, ESR, BASDAI, BASFI, ASDAS-CRP, ASDAS-ESR, HAQ, SF-36 physical and mental component scores (PCS and MCS), uveitis, inflammatory bowel disease, psoriasis, current use of NSAIDs and DMARDs, HLA-B27-positive, mNY-SI, MRI-SI, MRI-spine, low BMD, and DKK-1 level. Thereafter, we used multivariable analysis including in the model the variables with *p* < 0.15 on univariate analysis.

We conducted 2 analyses aiming to evaluate bone-related parameters: first the characteristics of the AxSpA patients associated with mSASSS score ≥ 1 and then with low BMD, including IL-31 serum level in the model. We performed as a first step univariate analysis including age, disease duration, sex, axial and peripheral involvement, tobacco use, CRP and hsCRP levels, ESR, BASDAI, BASFI, ASDAS-CRP, ASDAS-ESR, HAQ, SF36 PCS and MCS, uveitis, inflammatory bowel disease, psoriasis, current use of NSAIDs and DMARDs, HLA-B27-positive, mNY-SI, MRI-SI, MRI-spine, low BMD, DKK-1, sCD40L and IL-31. Thereafter, we used multivariable analysis including in the model only the variables with *p* < 0.15 on univariate analysis. Because IL-31 and sCD40L levels were highly correlated, we analyzed them separately in the multivariable models. All statistical analyses involved use of R software. Results were considered significantly different at p < 0.05.

## Results

### Characteristics of the population

The 708 patients included in the DESIR cohort had a mean age of 33.8 years; 51.5% were women, and 62.6% fulfilled the ASAS criteria. Serum levels were assessed in 2 subgroups of individuals at baseline: 1) patients from the DESIR cohort fulfilling the ASAS criteria (ASAS-positive, n = 443) and 2) healthy controls (51% men, mean age 32 ± 9.1 years) from the Variété cohort, age and sex-matched to a random sample of the DESIR cohort patients (control group, n = 79). Baseline characteristics of DESIR cohort ASAS+ patients are in Table 1.

**Table 1:** Characteristics of the study population (DESIR cohort ASAS+ patients).

Variables	ASAS-positive (n = 443)
**Clinics parameters**	
Age (mean ± SD)	31.43 ± 7.33 (n = 443)
Male, n (%)	215 (48.53%) (n = 443)
Disease duration (months) (mean ± SD)	18.85 ± 10.76 (n = 443)
Axial disease, n (%)	237 (53.50%) (n = 443)
Uveitis, n (%)	40 (33.06%) (n = 121)
IBD, n (%)	7 (1.58%) (n = 443)
Psoriasis, n (%)	72 (16.25%) (n = 443)
Enthesitis, n (%)	200 (45.15%) (n = 443)
Dactylitis, n (%)	58 (13.09%) (N = 443)
Synovitis, n (%)	13 (4.91%) (n = 442)
Tobacco use (ever vs never), n (%)	175 (39.59%) (n = 442)
**Biologic variables**	
HLA B27-positive, n (%)	373 (84.20%) (n = 443)
CRP (mean ± SD)	8.91 ± 13.70 (n = 428)
ESR (mean ± SD)	14.83 ± 16.70 (n = 427)
hsCRP (mean ± SD)	8.08 ± 14.05 (n = 434)
**Radiologic variables**, **n (%)**	
mSASSS ≥1	65 (15.37%) (n = 423)
mNY-SI-positive	121 (27.63%) (n = 438)
MRI-SI-positive	195 (46.54%) (n = 419)
MRI-spine-positive	86 (20.33%) (n = 423)
**BMD**, **n (%)**	
Low bone density	28 (13.08%) (n = 225)
**Current treatments**, **n (%)**	
NSAIDs	315 (71.11%) (n = 443)
Steroids	10 (2.26%) (n = 443)
DMARDs	43 (9.71%) (n = 443)
**Disease activity and handicap**, mean±SD	
BASDAI	42.53 ± 20.30 (n = 442)
BASFI	2.66 ± 1.02 (n = 363)
ASDAS-CRP	2.66 ± 1.02 (n = 363)

IBD, inflammatory bowel disease; HLA-B27, human leukocyte antigen B27; CRP, C-reactive protein; hsCRP, high-sensitivity CRP; ESR, erythrocyte sedimentation rate; BASDI, Bath Ankylosing Spondylitis Disease Activity Index; BASFI, Bath Ankylosing Spondylitis Functional Index; BASMI, Bath Ankylosing Spondylitis Metrology Index; BMD, bone mineral density; mSASSS, modified Stoke Ankylosing Spondylitis Spine Score; NSAIDs, nonsteroidal anti-inflammatory drugs; DMARDs, disease-modifying anti-rheumatic drugs.

### Increased serum levels of IL-31 and sCD40L in SpA

**Figure 1:**
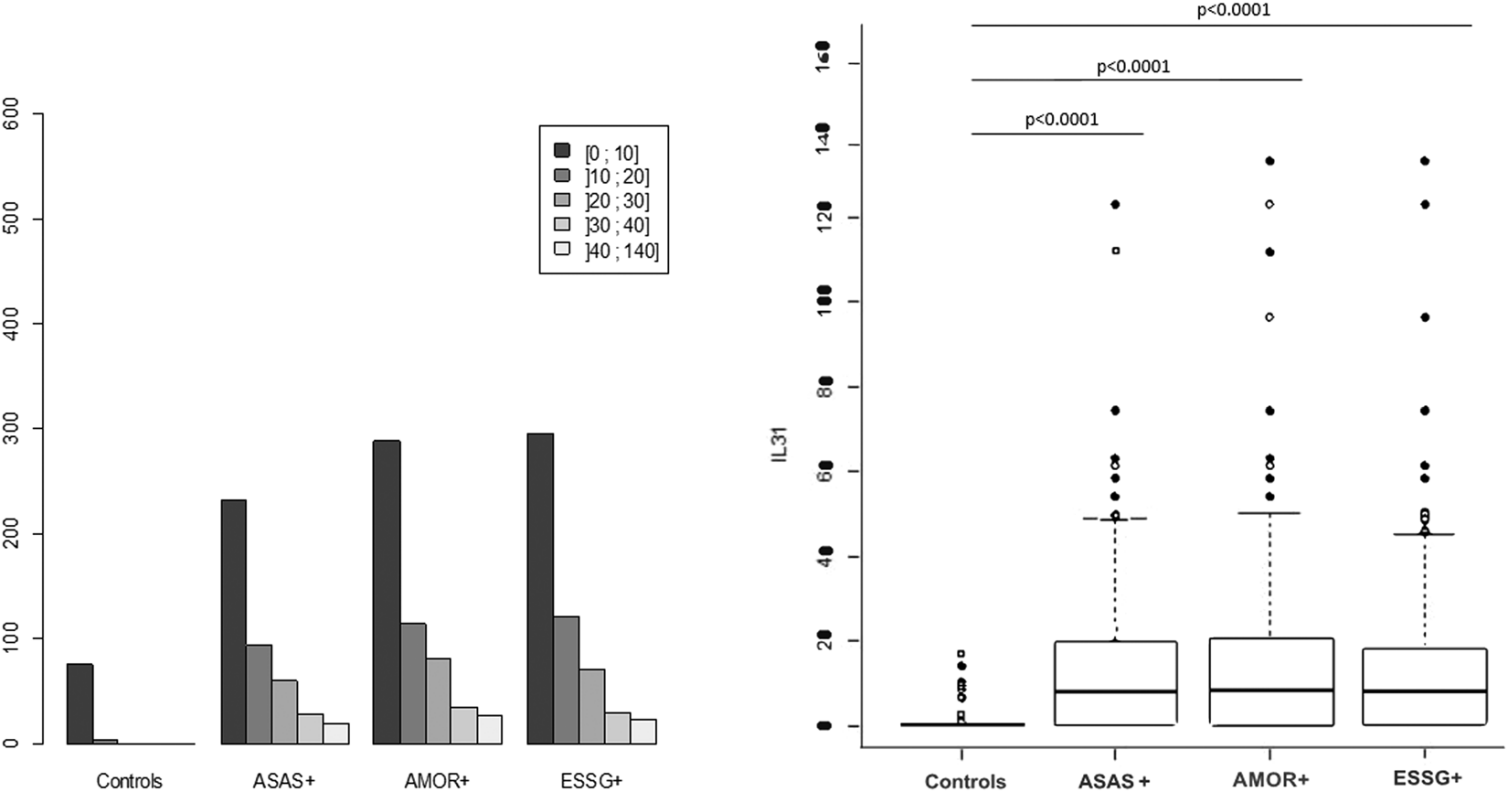
Distribution (left) and serum levels (right) of interleukin 31 (IL-31) among patients from the DESIR cohort who were positive for Assessment of Spondyloarthritis (ASAS), Amor (AMOR) and European Spondylarthropathy Study Group (ESSG) criteria and among healthy controls. (Right) Horizontal bar is median, box edges are Q1–Q3 and whiskers are ranges.

IL-31 serum levels distribution among controls and SpA patients according to the different classification criteria is presented in Fig. 1A. IL-31 was detectable among 60.9% of ASAS+ patients (n = 270) as compared with 18% of healthy controls (p < 0.0001) (Table 2). Quantification of the other cytokines is detailed in Table 2.

**Table 2:** Quantification of cytokines in ASAS+ patients and healthy control groups.

	ASAS+ (n = 443)			Controls (n = 79)		
	Detectable samples (%)	Range (pg/ml)	Median detectable (pg/ml)	Detectable samples (%)	Range (pg/ml)	Median detectable (pg/ml)
IL-31	270 (60.94%)	[0–122.88]	7.67	14 (17.72%)	[0–16.85]	0.00
sCD40L	419 (94.58%)	[0–1867.54]	463.33	75 (94.94%)	[0–1337.77]	318.70
TNFα	307 (69.30%)	[0–24.54]	1.88	76 (96.20%)	[0–10.41]	2.12
IL-6	268 (60.50%)	[0–124.20]	5.84	55 (69.62%)	[0–10.18]	0.99
IL-4	64 (14.44%)	[0–304.20]	12.00	12 (15.19%)	[0–114.41]	6.92
IL-33	30 (6.77%)	[0–860.50]	24.33	23 (29.11%)	[0–894.29]	84.07
DKK-1	434 (97.96%)	[0–134.48]	27.57	79 (100.00%)	[2.46–21.52]	11.27
IL-1β	112 (25.28%)	[0–30.62]	0.82	29 (36.71%)	[0–2.42]	0.16
IL-10	8 (1.81%)	[0–40.36]	16.49	7 (8.86%)	[0–37.64]	13.76
IL17-A	3 (0.67%)	[0–4.20]	2.73	4 (5.06%)	[0–2.34]	1.78
IL-17F	26 (5.86%)	[0–126.60]	34.16	5 (6.33%)	[0–119.75]	42.79
IL-21	7 (1.58%)	[0–184.80]	89.30	1 (1.27%)	[0–70.05]	70.05
IL-22	2 (0.45%)	[0–87.39]	46.70	2 (2.53%)	[0–9.37]	7.23
IL-23	5 (1.13%)	[0–267.3]	61.37	0 (0.00%)	—	—
IL-25	10 (2.26%)	[0–6.46]	1.71	3 (3.80)	[0–1.71]	1.71
IFNγ	6 (1.35%)	[0–44.35]	13.34	1 (1.27%)	[0–15.75]	15.75

The median value among quantifiable samples is provided as is the percentage of detectable samples.

Serum levels of IL-31 were higher for the ASAS+ than control group (mean 12.6 ± 15.4 pg/ml vs mean 1.8 ± 4.0 pg/ml; p < 0.0001) (Fig. 1). Increased IL-31 serum level was confirmed when using other classification criteria commonly used in SpA. In fact, IL-31 was significantly increased in the AMOR-positive group (n = 556) (mean 12.8 ± 16.2) versus the controls (p < 0.0001). The results were similar for the ESSG-positive group (mean 11.8 ± 15.2) versus the controls (p < 0.0001) (Fig. 1B).

Level of sCD40L, a T-lymphocyte activation biomarker^25^, was greater for ASAS-positive than control participants (mean 516.51 ± 349.8 pg/ml vs mean 412.43±318.45 pg/ml, p = 0.012). The 2 groups did not differ in serum levels of the cytokines IL-1β, IL-6, IL-10, IL-17A, IL-17F, IL-21, IL-22, IL-23, IFNγ, or TNFα (Table 2), nor in levels of the other Th2 investigated cytokines (IL-33, IL-4 and IL-25).

### Correlation of IL-31 with pro-inflammatory cytokines

We found a strong correlation between IL-31 and sCD40L levels (r = 0.6; p < 0.0001). The correlation with IL-6 and TNFα levels was weak but significant (r = 0.20; p < 0.001, and r = 0.23; p < 0.001, respectively). IL-31 level was correlated with DKK-1 level (r = 0.27; p = 0.0001) but not with the other studied cytokines (IL-1β, IL-10, IL-17A, IL-17F, IL-21, IL-22, IL-23, IL-25). These results were confirmed on multivariable analysis, finding an independent and significant association between IL-31 and sCD40L levels (p < 0.0001). We found no relevant correlation between IL-31 level and that of other Th2 cytokines known to be involved in IL-31 secretion: IL-4 (r = 0.13; p = 0.005) and IL-33 (r = −0.025; p = 0.59) (Suppl. Figure 1).

### Serum IL-31 level and clinical characteristics

IL-31 level was not associated with any of the patient characteristics of articular peripheral involvement (defined by peripheral painful joint), dactylitis, synovitis, enthesitis, uveitis, psoriasis, inflammatory bowel disease or HLA-B27 status. Also, NSAID or DMARD use was not associated with increased IL-31 level.

### Association of serum IL-31 level with disease activity in ASAS-positive patients

We found no relevant correlation between IL-31 level and the main acute-phase reactants among ASAS-positive patients (Suppl. Table 1). Similarly, we did not find any association of IL-31 serum level with disease activity or handicap scores (Suppl. Table 1). These results were confirmed by both univariate and multivariable analyses (data not shown).

### Association of increased IL-31 level with fewer structural lesions of the spine

**Figure 2:**
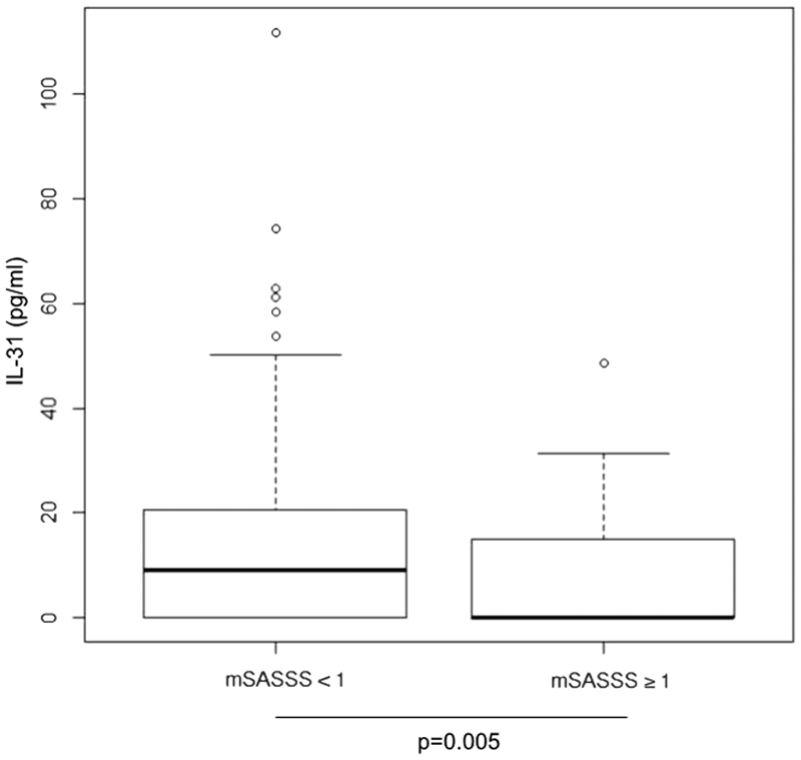
Levels of IL31 in mSASSS <1, and mSASSS ≥1 groups.

Higher serum level of IL-31 were observed in axSpA patients with mean mSASSS score <1: [mSASSS ≥ 1 (n = 65): 7.7 ± 10.7 vs mSASSS score <1 (n = 358) 13.02 ± 15.07, p = 0.0054] (Fig. 2).

No differences in serum IL-31 level were observed depending on the presence of radiographic sacroiliitis (mNY-positive (n = 121)14.0 ± 14.7 vs mNY-negative (n = 317) 11.6 ± 14.4; p = 0.09) nor inflammatory sacroiliitis seen on MRI (MRI-SI–positive (n = 195) 12.9 ± 15.6 vs MRI-SI–negative (n = 224) 12.3 ± 15.5; p = 0.58). Univariate analysis showed increased IL-31 level associated with mean mSASSS score <1 (p = 0.02) (Suppl. Table 2), which was confirmed by multivariable analysis (p = 0.03) (Suppl. Table 3).

We then assessed factors associated with structural damage (defined by mSASSS score ≥1) among ASAS+ patients. On univariate analysis, mSASSS score ≥1 was significantly associated with IL-31 level (p = 0.009) but not sCD40L level (p = 0.84) (Suppl. Table 4). Multivariable analysis further confirmed that each 10-pg/ml increase in IL-31 level protected against structural damage (odds ratio [OR] 0.948 [95% confidence interval [95% CI] 0.917–0.979], p = 0.001), while age, inflammation seen on spine MRI, and active disease assessed by ASDAS-ESR was associated with spinal structural lesions (Table 3).

**Table 3:** Multivariable analysis of factors associated with spinal structural lesions defined by mSASSS ≥1.

Variables	Odds ratio (95% CI)	P-value
Age	1.053 [1.007–1.101]	**0**.**0227**
hsCRP	0.992 [0.968–1.016]	0.5071
MRI Spine	4.522 [2.223–9.198]	<**0**.**0001**
ASDAS-ESR	1.654 [1.135–2.412]	**0**.**0089**
DKK1	1.010 [0.986–1.033]	0.4188
IL-31*	0.587 [0.917–0.979]	**0**.**0014**

^*^Odds ratio (95% CI) for each serum 10-pg/mL increase in IL-31 level.

We found the same statistically significant association for patients fulfilling the AMOR criteria (OR 0.674 [0.529–0.858], p = 0.0014) and the ESSG criteria (OR 0.738 [0.583–0.933], p = 0.011).

### Association of increased IL-31 level and low BMD

Both univariate and multivariable analyses showed IL-31 associated but not significantly with low BMD among the ASAS positive patients (p_trend_ = 0.058 and p_trend_ = 0.06, respectively) (Suppl. Tables 1 and 3).

*Briot et al*.^26^ has previously showed in the same cohort that 13.0% of patients had a low BMD and that the main risk factor associated with low BMD was inflammation on MRI. We then decided to assess factors associated with low BMD (defined by Z-score ≤ −2 SD) including IL-31 serum level in the model. On univariate analysis, among ASAS-positive AxSpA patients, gender (p = 0.001), enthesitis (p = 0.02), hsCRP level (p = 0.0001), Sacroilitis X-ray (p = 0.0002), MRI spine (p = 0.003) and ASDAS-ESR (p = 0.002) were significantly associated. Both IL-31 and sCD40L levels were associated but not significantly with low BMD (p = 0.11 and p = 0.06, respectively) (Suppl. Table 5).

On multivariable analysis, risk of low BMD was increased with each 10-pg/ml increase in IL-31 (OR 1.385 [95% CI 1.051–1.823], p = 0.01), together with male gender, enthesitis and MRI-SI-positivity (Table 4). Considering the high correlation between IL-31 and sCD40L levels, we further confirmed that sCD40L was not a confounding factor (Suppl. Table 6).

**Table 4:** Multivariable analysis of low BMD in ASAS-positive group.

Variables	Odds ratio (95% CI)	P-value
Male	6.738 [1.229–36.940]	**0**.**0280**
Enthesitis	3.847 [1.227–12.058]	**0**.**0208**
Hs CRP	1.002 [0.967–1.039]	0.9041
MRI SI-positive	2.375 [0.688–8.199]	0.1711
Sacroilitis X-ray	3.229 [0.846–12.231]	0.0863
MRI Spine	1.248 [0.318–4.896]	0.7504
ASDAS ESR	2.100 [0.958–4.603]	0.0639
BASFI	0.964 [0.711–1.308]	0.8149
IL-31*	1.367 [1.007–1.855]	**0**.**0449**

^*^Odds ratio for each 10-pg/mL increase in IL-31 level.

In the AMOR-positive group, risk of low BMD was increased with each 10-pg/ml increase in IL-31 (OR 1.335 [95% CI 1.050–1.697], p = 0.018). In the ESSG-positive group, the results for low BMD were not significant (OR 1.189 [0.834–1.695], p = 0.34).

## Discussion

This study, involving a large cohort of young adults with early IBP suggestive of SpA found 61% of patients from the ASAS-positive of the DESIR cohort with increased level of IL-31 as compared with only 18% in the control group. Increased serum level of IL-31 was significantly associated with less structural damage but also low BMD, which suggests that IL-31 might be involved in the bone formation/resorption balance in AxSpA. The lack of a statistically significant association of IL-31 with acute-phase reactants despite a statistically significant correlation with sCD40L level suggests that this effect could be independent of inflammation but could rely on T-cell activation.

IL-31 and sCD40L were the only 2 cytokines from the 17-plex ELISA showing increased levels in the DESIR cohort. Levels of the other studied cytokines, especially Th17 cytokines, were not significantly increased, despite the use of a Luminex technology that is usually considered quite sensitive. These results suggest that 1) serum Th17 cytokines may poorly reflect the level of activation of this pathway in AxSpA, at least at an early stage of the disease; 2) Th17 cytokines producing cells in peripheral blood mononuclear cells may be in a steady state, expressing low serum levels of cytokines that should be quantified in cell-stimulation conditions; and 3) Luminex assays, even if considered sensitive to assess cytokine serum levels may not be sensitive enough to quantify Th17 cytokines in early AxSpA.

IL-31 is a four-bundle helix cytokine belonging to the gp130/IL-6 cytokine family. It binds an heterodimeric receptor consisting of the IL-31 receptor A (IL-31RA) and the Oncostatin M receptor (OSMR). This receptor can recruit 3 signaling pathways: the Jak/STAT pathway, the PI3K/AKT pathway and the MAPK pathway^27–29^. The 2 subunits of the receptor are preferentially expressed in skin, testis, bone marrow, brain and thymus for IL-31RA^28,30,31^ and is more ubiquitous in humans organs for OSMR^28,31^. Upon stimulation, monocytes, macrophages, dendritic cells and mast cells are also able to produce IL-31.

IL-31 level was strongly correlated with level of sCD40L, a T-cell activation marker. The biological effect of IL-31 is currently unclear, and its involvement in some inflammatory processes is controversial. In 2004, Dillon *et al*. reported for the first time the expression of IL-31RA in bone marrow and the ability of IL-31 to induce Th1 polarization under specific conditions^30^. Subsequently, various conflicting data emerged: IL-31 could limit Th2-inflammation in the lung^32^ and the intestine^33^ but could have a pro-inflammatory effect in the skin^34–36^. Yagi *et al*. reported that in human colonic sub-epithelial myofibroblasts, the effect of IL-31 on the secretion of cytokines, chemokines and matrix metalloproteinases was similar to IL-17A^37^, so IL-31 might be a Th17-related cytokine. Several groups have reported an elevation of IL-31 in skin from patients with atopic dermatitis, in bronchoalveolar lavage fluid from patients with allergic asthma, and rhinitis; in intestinal mucosa from patients with inflammatory bowel disease; and recently in sera and aqueous humour from patients with acute anterior uveitis associated with HLA-B27 and osteoporosis^27,34,35,38–44^.

We observed a significant correlation between IL-31 and DKK-1 levels. A recent publication^45^ reported that DKK-1 polarizes CD4+ T cells to the Th2 cell lineage. DKK-1 may favour a Th2 response in SpA patients, consequently associated with increased IL-31. Nevertheless, levels of other cytokines (IL-4 and IL-10), usually considered surrogate markers for Th2 polarization, were not increased in AxSpA patients from the DESIR cohort. However, Notani *et al*. proposed that high concentrations of DKK-1 or Wnt3a could inhibit Th2 differentiation^46^. Considering that one third of AxSpA patients have high serum levels of DKK-1^24^, the Th1/Th2 polarization induced by DKK-1 might be equivocal in AxSpA. In fact, in our study, IL-31 level was rather consistently correlated with levels of pro-inflammatory cytokines such as IL-6 or TNFα. These data support that IL-31 may have a role in SpA and more generally in the Th1/Th2 balance of the disease in a context of high levels of DKK-1 expression. IL-31 appears to be a cytokine at the border of the Th1 and Th2 polarization. Our data suggest that activated T cells could be the source of IL-31 production, but mast cells are also another source of IL-31. This latter cell type seems to play an important role in SpA pathogenesis via secretion of IL-17^47^, but its role as an IL-31–secreting cell could also be further assessed.

To the best of our knowledge, this is the first report suggesting a potential role for IL-31 in low BMD and absence of structural damage in SpA. Indeed, patients with low BMD had high levels of IL-31. An increase in IL-31 serum levels was previously observed in post-menopausal osteoporosis^44^. Nevertheless, the accurate role of IL-31 in bone remodelling is currently an unexplored field. IL-31 may be involved in the activation of the RANK/RANKL pathway or alternatively act by inhibiting Wnt signaling. This suggestion could be consistent with our analysis, because IL-31 level was correlated with serum DKK-1 level. In patients with ankylosing spondylitis, syndesmophyte formation was reduced with high functional DKK-1 level, which suggests that blunted Wnt signaling suppresses new bone formation and thereafter syndesmophyte growth in ankylosing spondylitis^48^.

Our study had some limitations. Although the Luminex technology does not have the highest sensitivity to quantify cytokines, our results show a significant difference in IL-31 and sCD40L levels between healthy controls and SpA patients in a large sample. Moreover, unlike Th17 cytokines, IL-31 level was increased at a steady state without any stimulation. Such lack of cell stimulation probably explains why we did not find an increase in serum levels of Th1 and Th17 cytokines. Although the initial cohort was large, the proportion of patients with low BMD was low; thus, the relationship between serum level of IL-31 and the measurement of BMD should be interpreted with caution. As for all cross-sectional studies, causality cannot be ascertained with our observations. These results must be confirmed with prospective data. Lack of centralized quality control of BMD measurements (i.e., use of different devices, absence of cross-calibration) and the low sample size of patients with BMD measurements is a limitation of our cross-sectional study; however, participating centers had expertise in BMD measurements and followed the recommendations for quality control of the device.

To conclude, our study, performed in a large cohort of patients, highlights the potential participation of IL-31 in the pathogenesis of structural damage in AxSpA. Our data support that IL-31 may participate in the bone remodelling processes responsible for the disease. We demonstrated an association of high IL-31 level with less structural damage in the spine and with the osteoporotic/osteopenic phenotype of patients. IL-31 may be involved in the bone loss found in SpA patients. The reason for the increased IL-31 level in AxSpA has yet to be determined. These data need to be confirmed in the most severe forms of the disease and assessed among AxSpA patients with longer disease duration. Nevertheless, these findings highlight new lines of thought about the pathophysiology of the structural changes in this disease, especially regarding the role of IL-31 in bone formation and could open new therapeutic perspectives.

## Electronic supplementary material

Supplementary information
