# Supplementary material for: Increase In Il-31 Serum Levels Is Associated With Reduced Structural Damage In Early Axial Spondyloarthritis
Source: Sci Rep. 2018 May 16;8:7731. doi: 10.1038/s41598-018-25722-z (PMC5956108; doi:10.1038/s41598-018-25722-z)
Supplement: Supplementary file 1 — Supplementary information [file 41598_2018_25722_MOESM1_ESM.pdf]

INCREASE IN IL-31 SERUM LEVELS IS ASSOCIATED WITH REDUCED STRUCTURAL DAMAGE IN EARLY AXIAL SPONDYLOARTHRITIS

Data from the DESIR Cohort

Nicolas Rosine[1], Adrien Etcheto[1,2], Houria Hendel-Chavez[3], Raphaèle Seror[4], Karine Briot[1,2], Anna Molto[1,2], Philippe Chanson[5], Yassine Taoufik[3], Daniel Wendling[6], Rik Lories[7], Francis Berenbaum[8], Rosaline van den Berg[9], Pascal Claudepierre[10], Antoine Feydy[11], Maxime Dougados[1, 2, 12], Christian Roux[1,2], Corinne Miceli-Richard[1,12]

Variables	Mean (sd)	Correlation r	p-value
CRP	8.9 (13.7)	0.184	<0.0001
ESR	14.8 (16.7)	0.098	0.0443
Hs CRP	8.1 (14.0)	0.116	0.0158
BASDAI	42.5 (20.3)	0.001	0.9968
BASFI	2.9 (2.2)	0.019	0.6890
BASMI	1.9 (1.1)	-0.004	0.4235
ASDAS CRP	2.7 (1.0)	0.089	0.0961
ASDAS ESR	2.4 (1.0)	0.004	0.4590
HAQ-AS	0.56 (0.49)	0.001	0.7494
SF36 PCS	40.5 (9.2)	0.023	0.6353
SF36 MCS	40.9 (11.3)	-0.048	0.3178

Suppl.Table 1: correlations of IL-31 with acute phase reactants and disease activity and handicap scores
CRP, C-reactive protein; ESR, erythrocyte sedimentation rate; hsCRP, high-sensitivity CRP; BASDAI, Bath Ankylosing Spondylitis Disease Activity Index; BASFI, Bath Ankylosing Spondylitis Functional Index; BASMI, Bath Ankylosing Spondylitis Metrology Index; ASDAS, Ankylosing Spondylitis Disease Activity Score; HAQ-AS, Health Assessment Questionnaire for Ankylosing Spondylitis; SF-36, short form 36; PCS, Physical Component Summary; MCS, Mental Component summary.

Variables	coefficient	p-value
Age	-0.033	0.7478
Disease duration	-0.003	0.9587
Male	1.435	0.3328
Ever smoking	-1.025	0.4950
Articular peripheral involvement	0.227	0.8783
Uveitis	-5.533	0.1047
IBD	-0.387	0.9475
Psoriasis	0.0950	0.9622
HLAB27+	-3.356	0.1012
CRP	0.113	0.0409
ESR	0.095	0.0253
Hs CRP	0.108	0.0411
mNY-SI	2.414	0.1668
MRI-SI	0.584	0.7048
MRI spine	0.703	0.7119
mSASSS ≥ 1	-1.17	0.0208
Average Syndesmophyte	-2.55	0.0276
Low BMD	5.081	0.0582
BASDAI	0.001	0.9783
BASFI	-0.1166	0.7277
BASMI	-0.635	0.3318
ASDAS CRP	1.257	0.1077
ASDAS ESR	0.7485	0.3041
HAQ	-0.037	0.9803
SF36 PCS	0.047	0.5544
SF36 MCS	-0.013	0.8418
Current use of oral NSAIDs	0.899	0.5824
Current use of DMARDs	-3.452	0.1635
DKK1	0.212	<0.0001
CD40L	0.025	<0.0001
Low BMD	5.081	0.0582

Suppl Table 2 : Univariate analysis IL-31 level in ASAS-positive group (p<0.15)
IBD, inflammatory bowel disease; HLA-B27, human leukocyte antigen B27; CRP, C-reactive protein; ESR, erythrocyte sedimentation rate; hsCRP, high-sensitivity CRP; mNY-SI, X-ray of sacroiliac joints; MRI-spine, MRI of the whole spine; MRI-SI, MRI of sacroiliac joints; BASDAI, Bath Ankylosing Spondylitis Disease Activity Index; BASFI, Bath Ankylosing Spondylitis Functional Index; BASMI, Bath Ankylosing Spondylitis Metrology Index; ASDAS, Ankylosing Spondylitis Disease Activity Score; HAQ-AS, Health Assessment Questionnaire for Ankylosing Spondylitis; SF-36, short form 36; PCS, Physical Component Summary; MCS, Mental Component summary; mSASSS, modified Stoke Ankylosing Spondylitis Spine Score; NSAIDs, nonsteroidal anti-inflammatory drugs; DMARDs, disease-modifying anti-rheumatic drugs; BMD, bone mineral density.

Variables	Coefficient	P-value
Hs CRP	0.015	0.8537
mSASSS ≥ 1	-5.939	0.0353
Low BMD	6.625	0.0634
HLA B27+	0.983	0.7538
ASDAS CRP	-0.835	0.5054
DKK1	0.185	0.0857
sCD40L	0.020	<0.0001

Suppl Table 3 : Multivariate analysis parameters associated with IL-31
hsCRP, high-sensitivity CRP; mSASSS, modified Stoke Ankylosing Spondylitis Spine Score; BMD, bone mineral density; HLA-B27, human leukocyte antigen B27; ASDAS, Ankylosing Spondylitis Disease Activity Score.

Variables	Odds ratio (95% CI)	P-value
**Clinical variables**		
Age	1.050 [1.012–1.089]	**0.0101**
Male	1.392 [0.815–2.376]	0.2260
Disease duration	1.008 [0.984–1.033]	0.4996
Peripheral articular involvement	0.959 [0.565–1.628]	0.8765
Uveitis	0.798 [0.263–2.416]	0.6892
IBD	0.917 [0.109–7.742]	0.9363
Psoriasis	0.885 [0.427–1.833]	0.7428
Enthesitis	1.262 [0.743–2.142]	0.3888
Tobacco use	1.347 [0.790–2.297]	0.2745
**Biologic variables**		
HLA-B27-positive	0.710 [0.362–1.393]	0.3194
CRP level	1.010 [0.993–1.027]	0.2616
ESR	1.011 [0.997–1.026]	0.1114
hsCRP level	1.016 [1.000–1.031]	**0.0495**
**Radiologic variables**		
mNY-SI	1.450 [0.823–2.556]	0.1984
MRI-SI	1.397 [0.812–2.402]	0.2274
MRI Spine	1.092 [1.048–1.137]	**<0.0001**
Low bone density	2.288 [0.918–5.702]	0.0757
Treatments		
Current use of NSAIDs	1.018 [0.570–1.820]	0.9511
Use of steroids	1.147 [0.548–2.399]	0.7165
Current use of DMARDs	1.150 [0.487–2.718]	0.7500
Disease activity		
BASDAI	1.013 [1.000–1.027]	**0.0499**
BASFI	1.076 [0.958–1.209]	0.2159
ASDAS-CRP	1.253 [0.959–1.655]	0.1117
ASDAS-ESR	1.355 [1.033–1.776]	**0.0280**
HAQ week	1.444 [0.863–2.416]	0.1613
SF-36 PCS	0.992 [0.964–1.021]	0.5888
SF-36 MCS	0.999 [0.976–1.023]	0.9600
DKK1 level	1.013 [0.998–1.028]	0.1016
sCD40L level	0.971 [0.737–1.281]	0.8370
IL-31 level	0.718 [0.559–0.921]	0.0092

Suppl Table 4 : Univariate analysis for mSASSS ≥ 1

IBD, inflammatory bowel disease; HLA-B27, human leukocyte antigen B27; CRP, C-reactive protein; hsCRP, high-sensitivity CRP; ESR, erythrocyte sedimentation rate; mNY-SI, X-ray of sacroiliac joints; MRI-spine, MRI of the whole spine; MRI-SI, MRI of sacroiliac joints; BMD, bone mineral density; mSASSS, modified Stoke Ankylosing Spondylitis Spine Score; NSAIDs, nonsteroidal anti-inflammatory drugs; DMARDs, disease-modifying anti-rheumatic drugs; BASDAI, Bath Ankylosing Spondylitis Disease Activity Index; BASFI, Bath Ankylosing Spondylitis Functional Index; ASDAS, Ankylosing Spondylitis Disease Activity Score; HAQ-AS, Health Assessment Questionnaire for Ankylosing Spondylitis; SF-36, short form 36; PCS, Physical Component Summary; MCS, Mental Component summary.

Variables	Odds Ratio	P-value
Clinics parameters		
Age	0.992 [0.940 – 1.048]	0.7847
Male	7.390 [2.161 – 25.264]	0.0014
Disease duration	1.000 [0.966 – 1.035]	1.0000
Articular peripheral involvment	1.328 [0.601 – 2.934]	0.4837
Uveitis	1.000 [0.268 – 3.732]	1.0000
Psoriasis	1.750 [0.687 – 4.459]	0.2409
Enthesitis	2.631 [1.170 – 5.921]	0.0194
Tobacco	1.508 [0.680 – 3.344]	0.3121
Biologics parameters		
HLA B27+	0.780 [0.275 – 2.216]	0.6410
CRP	1.049 [1.026 – 1.072]	<0.0001
ESR	1.046 [1.024 – 1.068]	<0.0001
HsCRP	1.043 [1.021 – 1.065]	0.0001
Radiologics parameters		
mNY-SI	4.870 [2.112 – 11.228]	0.0002
MRI-SI	3.047 [1.261 – 7.362]	0.0133
MRI-Spine	1.088 [1.028 – 1.151]	0.0003
Treatments		
Current use of NSAID	0.948 [0.395 – 2.279]	0.9055
Use of steroids	1.825 [0.677 – 4.923]	0.2347
Current use of DMARDs	1.917 [0.592 – 6.209]	0.2780
Disease activity		
BASDAI	1.012 [0.993 – 1.031]	0.2309
BASFI	1.151 [0.975 – 1.360]	0.0970
ASDAS-CRP	1.987 [1.299 – 3.040]	0.0016
ASDAS-ESR	1.900 [1.271 – 2.841]	0.0018
HAQ week	1.539 [0.724 – 3.371]	0.2629
Score PCS	0.991 [0.950 – 1.034]	0.6781
Score MCS	0.980 [0.946 – 1.016]	0.2808
DKK1	1.004 [0.974 – 1.035]	0.7992
sCD40L	1.418 [0.980 – 2.052]	0.0640
IL-31	1.198 [0.958 – 1.497]	0.1130

Suppl Table 5: Univariate analysis low BMD in ASAS-positive group

IBD, inflammatory bowel disease; HLA-B27, human leukocyte antigen B27; CRP, C-reactive protein; hsCRP, high-sensitivity CRP; ESR, erythrocyte sedimentation rate; mNY-SI, X-ray of sacroiliac joints; MRI-spine, MRI of the whole spine; MRI-SI, MRI of sacroiliac joints; BMD, bone mineral density; mSASSS, modified Stoke Ankylosing Spondylitis Spine Score; NSAIDs, nonsteroidal anti-inflammatory drugs; DMARDs, disease-modifying anti-rheumatic drugs; BASDAI, Bath Ankylosing Spondylitis Disease Activity Index; BASFI, Bath Ankylosing Spondylitis Functional Index; ASDAS, Ankylosing Spondylitis Disease Activity Score; HAQ-AS, Health Assessment Questionnaire for Ankylosing Spondylitis; SF-36, short form 36; PCS, Physical Component Summary; MCS, Mental Component summary.

Variables	Odds ratio (95% CI)	P-value
Male	7.582 [1.381 – 41.634]	0.0197
Enthesitis	3.666 [1.181 – 11.381]	0.0246
hsCRP	1.002 [0.968 – 1.037]	0.9208
mNY-SI	4.279 [1.116 – 16.400]	0.0339
MRI-SI	1.940 [0.582 – 6.464]	0.2803
MRI-Spine	0.910 [0.241 – 3.432]	0.8887
ASDAS ESR	2.146 [0.986 – 4.668]	0.0542
BASFI	0.948 [0.705 – 1.275]	0.7230
sCD40L level	1.207 [0.732 – 1.990]	0.4610

Suppl Table 6: Multivariate analysis of low BMD in ASAS-positive group, assessing the contribution of sCD40l

*Odds ratio for each 1-SD increase in sCD40L level (SD=344.81 pg/ml)

hsCRP, high-sensitivity CRP; mNY-SI, X-ray of sacroiliac joints; MRI-spine, MRI of the whole spine; MRI-SI, MRI of sacroiliac joints; ASDAS, Ankylosing Spondylitis Disease Activity Score; BASFI, Bath Ankylosing Spondylitis Functional Index.

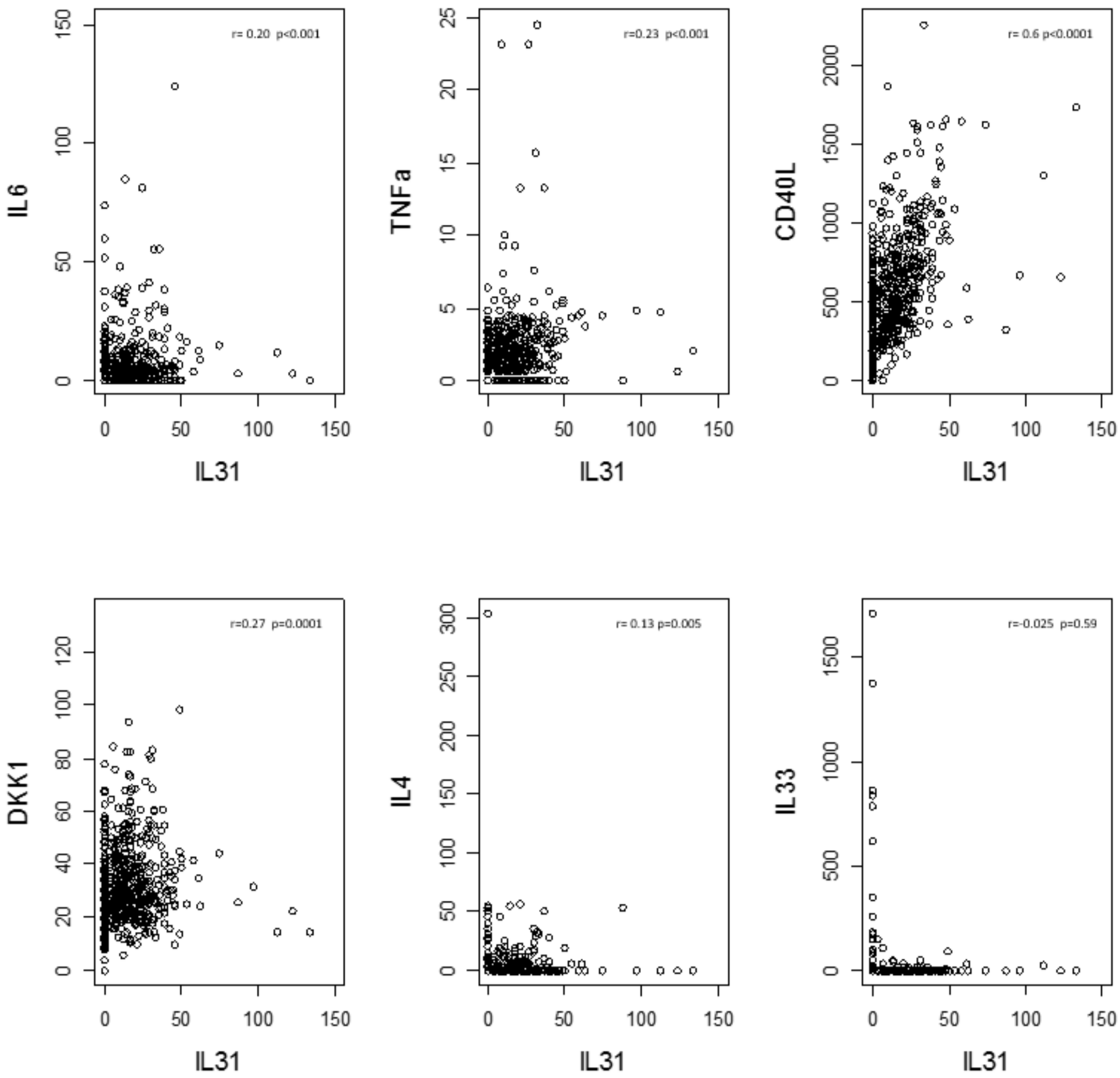

Suppl. Figure 1: Correlation analysis of serum levels of IL-31 and IL-6, tumor necrosis factor α (TNFα), sCD40L, DKK-1, IL-4, and IL-33.
